# Supplementary material for: SARS-CoV-2 in severe COVID-19 induces a TGF-β-dominated chronic immune response that does not target itself
Source: Nat Commun. 2021 Mar 30;12:1961. doi: 10.1038/s41467-021-22210-3 (PMC8010106; doi:10.1038/s41467-021-22210-3)
Supplement: Supplementary file 1 — Supplementary Information [file 41467_2021_22210_MOESM1_ESM.pdf]

Supplementary Information

SARS-CoV-2 in severe COVID-19 induces a TGF-β-dominated chronic immune response that does not target itself

Marta Ferreira-Gomes *et al.*

Corresponding author: Mir-Farzin Mashreghi, mashreghi@drfz.de

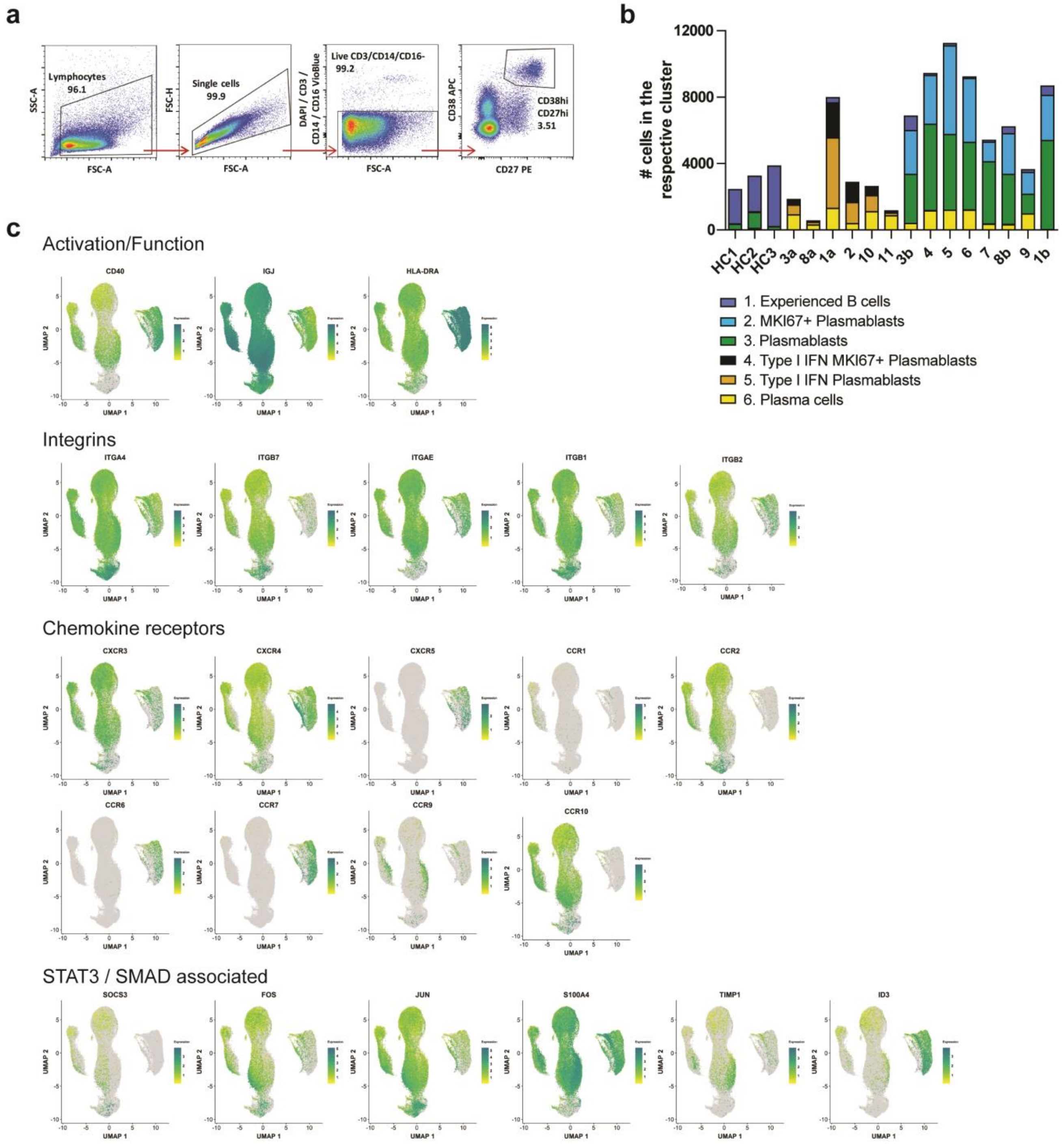

Supplementary Figure 1. Single cell transcriptomes of CD38^{high} CD27^{high} cells from ICU COVID-19 patients and healthy controls. a) Gating strategy used for FACS of activated/differentiated B cells (CD27^{high} CD38^{high}) from fresh peripheral blood of COVID-19 patients and healthy controls depicted in Figure 1. Before FACS, cells were pre-enriched by MACS using StraightFrom blood CD19 MicroBeads. **b)** Number of cells per donor present in each cluster. **c)** UMAP representation of the expression levels of selected activation/function, integrin, chemokine receptor and IL-21/STAT3 and TGF-β/SMADs signaling associated genes of sorted CD27^{high} CD38^{high} B cells from 9 COVID-19 ICU patients and 3 healthy controls (see Fig. 1b).

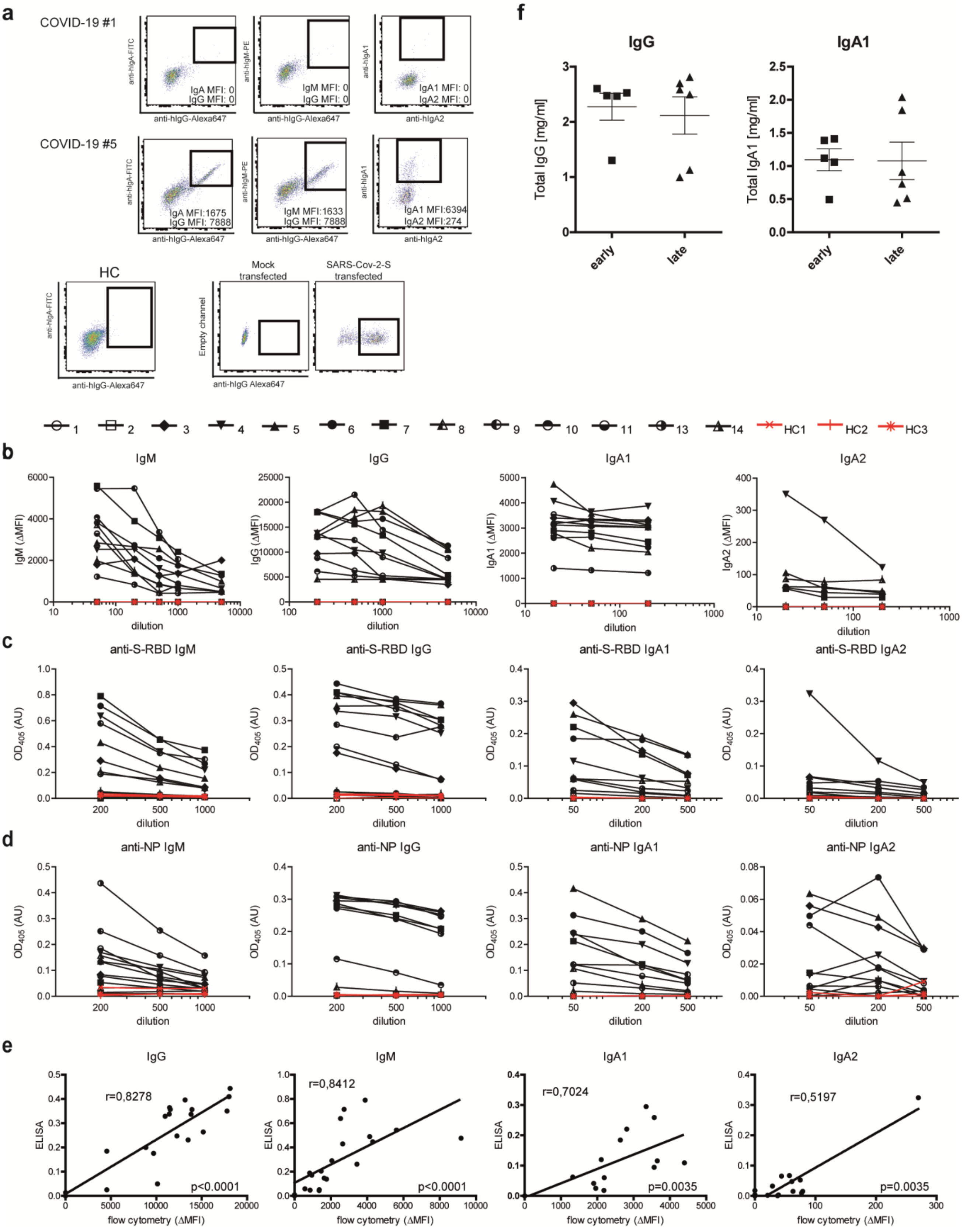

Supplementary Figure 2. Serology of ICU COVID-19 patients and healthy controls. a) Profiles of serum antibodies specific for SARS-Cov-2 S protein of COVID-19 patients. Representative dot plots of viable 293T cells transfected with either a SARS-CoV-2 S protein-expressing plasmid or mock transfected, incubated either with the recombinant anti-spike protein antibody (CR3022), as a control, or with serum (diluted 1:50 in PBS) of COVID-19

patients and healthy controls, and stained with fluorescently labelled anti-hIgG, anti-hIgA-FITC, anti-hIgM-PE, anti-hIgA1 or anti-hIgA2. **b)** Binding of IgG, IgM, IgA1 and IgA2 antibodies to SARS-CoV-2-S protein expressed on the surface of transfected 293T cells. Data represent ΔMFI, which was calculated as following: MFI (transfected cells, incubated with sera and stained with respective anti-hIgA1 or anti-hIgA2) – MFI (untransfected cells, incubated with sera and stained with respective anti-hIgA1 or anti-hIgA2). **c**) Binding of IgG, IgM, IgA1 and IgA2 antibodies to SARS-CoV-2-S-RBD protein in COVID-19 ICU patients quantified by ELISA. When more than one time point per patient was available, the earliest time point was used. **d)** Binding of IgG, IgM, IgA1 and IgA2 antibodies to SARS-CoV-2-NP in COVID-19 ICU patients quantified by ELISA. When more than one time point per patient was available, the earliest time point was used. **e)** Correlation between SARS-CoV-2 S-RBD antibodies measured by ELISA versus antibodies binding to the Sars-CoV-2 S protein expressed on the surface of 293 T cells. Correlations for specific IgG and IgM antibodies were performed using sera from COVID-19 patients diluted 200 times, for IgA1 and IgA2 – sera was diluted 50 times. Spearman rank correlation was applied for analysis of correlations between ELISA- and flow cytometry- based antibody measurements. **f)** Concentration of total IgG and IgA1 in the serum of COVID-19 patients at early (less than a week) and late time points after ICU admission as determined by ELISA.

a

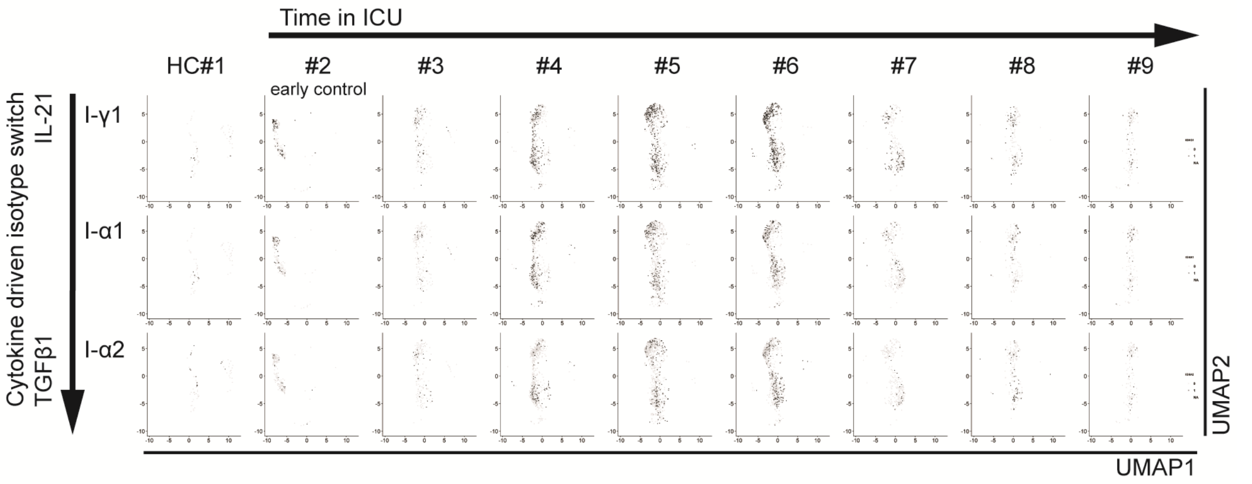

b

Active Allele (columns); Inactive Allele (rows)

COVID-19	IGHA1	IGHA2	IGHG1	IGHG2	IGHG3	IGHG4	IGHD	IGHE	IGHM
IGHA1	13	9	20	21	25	10	4	6	24
IGHA2	18	15	5	34	8	5	2	0	6
IGHG1	29	22	19	7	10	24	22	0	34
IGHG2	7	21	6	12	2	4	12	0	4
IGHG3	6	3	1	1	9	35	12	0	2
IGHG4	0	6	0	0	0	6	2	0	1
IGHD	0	0	0	1	0	0	0	0	0
IGHE	0	0	0	0	0	0	0	0	0
IGHM	27	23	48	24	35	4	23	6	30

c

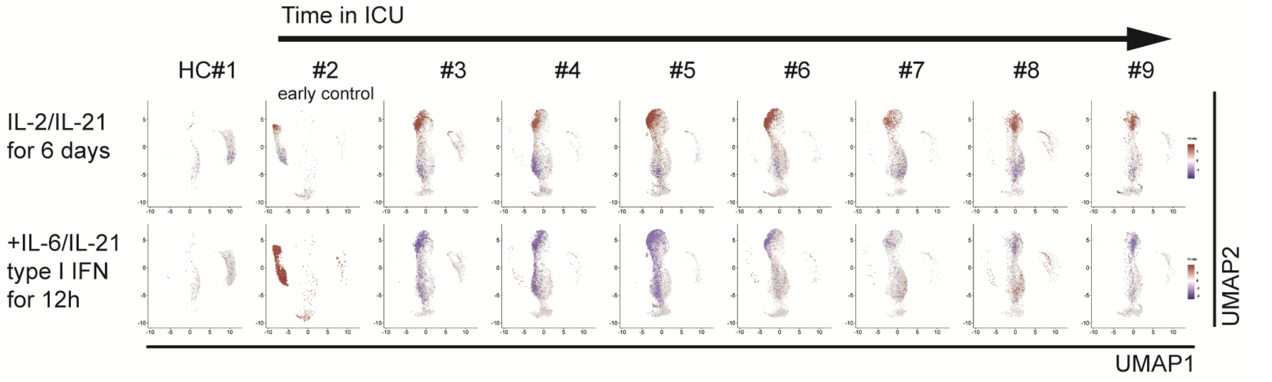

Supplementary Figure 3. Class switch recombination of plasmablasts from COVID-19 patients and healthy controls. a) UMAP representation of the expression of the Fc region of the inactive heavy Ig chain for individual cells transcribing two Ig heavy chain gene loci. Along with an active Ig heavy chain B cells can transcribe the inactive, allelically excluded, nonproductive IgH locus, starting from pseudoexons (I exons) before the switch regions, under the control of distinct cytokines as discussed above, and proceeding through the entire adjacent Ig constant region gene (germline/switch transcripts). Arrows represent the time patients had spent in intensive care at the time of analysis (upper) and the shift in cytokine milieu to allow switching to IgG1/IgA1/IgA2 (left). **b)** Correlation of Fc regions of the active and inactive heavy chain transcripts for COVID-19 patients. Shown are cell counts with identified two heavy chain transcripts: an active productive transcript, showing up to 100x higher expression and an inactive nonproductive IgH transcript. **c)** Gene Set Enrichment Analysis (GSEA) for IL-2/IL-21 and IL-6/IL-21/type I IFN-stimulation signature genes as defined by contrasting IL-2/IL-21-activated B cells and IL-2/IL-21-activated B cells with an additional IL-6/IL-21/type I IFN, IL-6/IL-21/TGF-β or IL-6/IL-21/type I IFN/TGF-β treatment for 12h. Signature genes were based on the gene expression data set published by Stephenson

et al.[16] where peripheral blood B cells from healthy donors were differentiated into plasmablasts in presence of IL-2 and IL-21, and subsequently stimulated with different cytokine cocktails during different time periods. GSEA was performed for each cell relative to the mean expression in COVID-19 and healthy control samples. Cells with statistically significant enrichments were color-scaled by the normalized enrichment score (NES), with red shades for cells showing up- and blue for cells showing down-regulation. UMAP was performed collectively with all COVID-19 and healthy samples and separated for visualization.

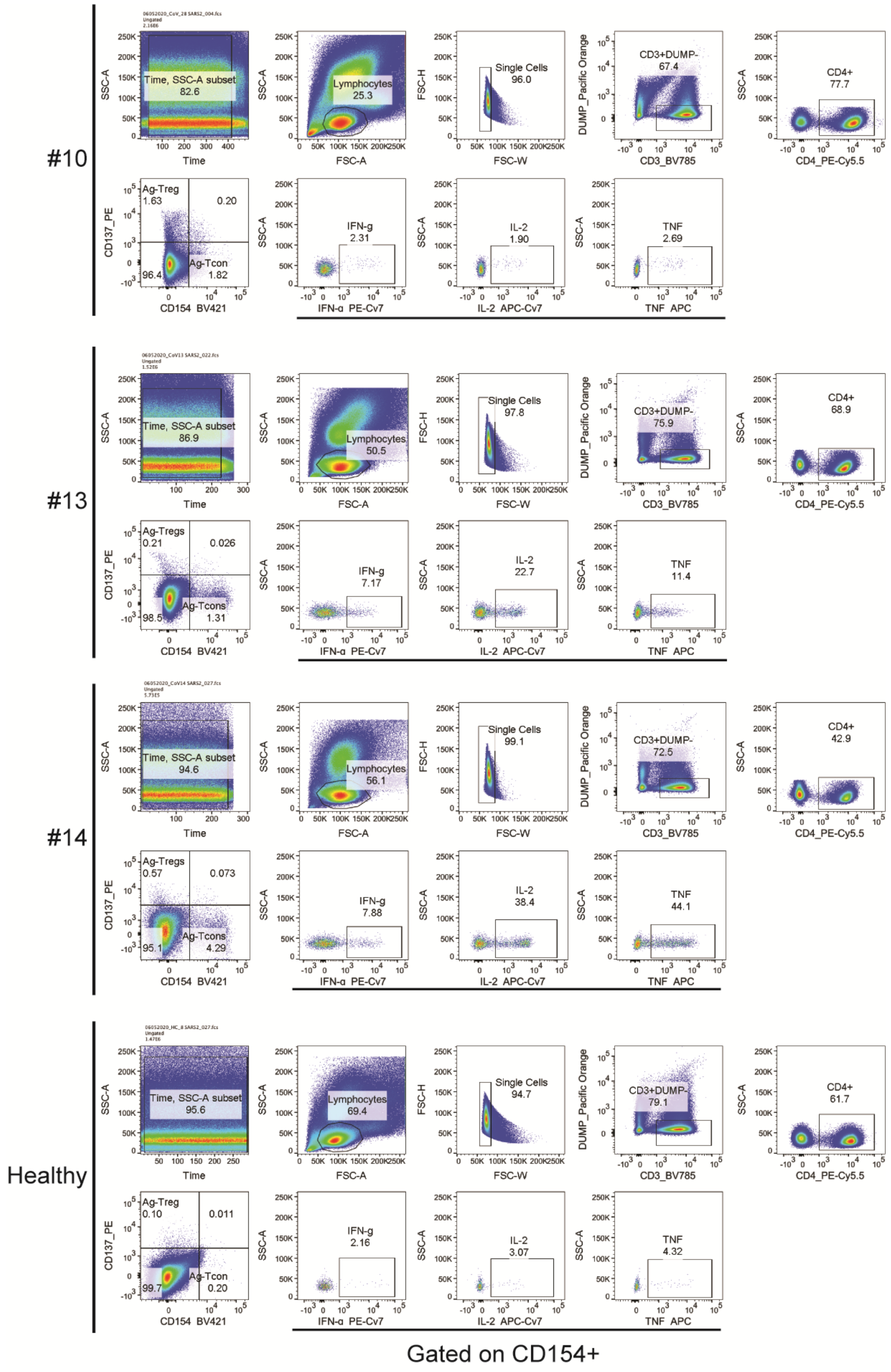

Supplementary Figure 4. Flow cytometric analysis of SARS-CoV-2-reactive peripheral blood CD4$^+$ T cells. For analysis, PBMCs from 3 COVID-19 ICU patients and 1 healthy control were stimulated for 6 hours with anti-CD28 and a mixed peptide pool representing the SARS-CoV-2 spike glycoprotein (S), the membrane glycoprotein (M), and the nucleocapsid phosphoprotein (N) in presence of anti-CD40. Antigen-reactive regulatory T cells were identified by the upregulation of CD137, while antigen-reactive effector T cells were identified by the upregulation of CD154. This gating strategy was also used for cytometric sorting of CD137$^+$ and CD154$^+$ CD4$^+$ T cells for single cell transcriptomics and repertoire analysis depicted in Figure 4. Staining for IFN-γ, IL-2 and TNF gated on CD4$^+$CD154$^+$ T cells.

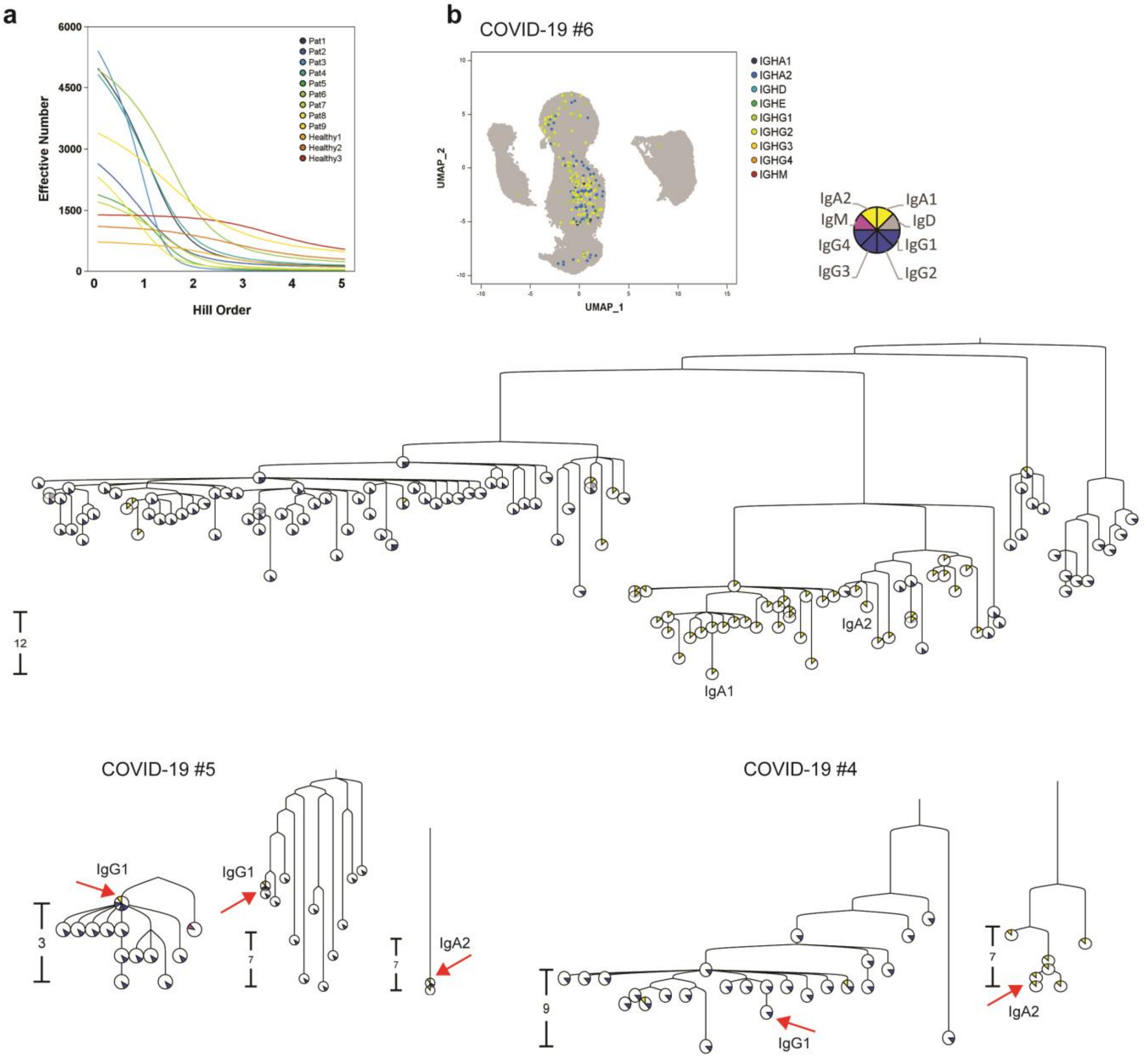

Supplementary Figure 5. Oligoclonality and somatic hypermutation of the antigen-receptor repertoire of plasmablasts from COVID-19 patients. a) Effective number of clones (diversity) of individual patients at different Hill orders. Hill order of 0 represents the observed number of different clonotypes. **b)** Clonal relatives of 6 selected clonal families from COVID-19 patients 6 (clone distribution in UMAP projection of activated B cell clusters and clonal tree), 5 and 4 (clonal trees only). A clonal family comprise clones with the same germline FR1-FR3 sequence, used V-, J- genes and the length of the cdr3 region in both heavy and light chain. Each pie chart represents at least one clone with a particular FR1-FR3 sequence. Numbers indicate the nucleotide differences (mutations) discriminating cells from their next common ancestor. Marked by red arrows are clones, from which the reconstructed BCR sequences were used to produce recombinant antibodies. These Antibodies were tested for binding to S and NP of SARS-CoV-2.

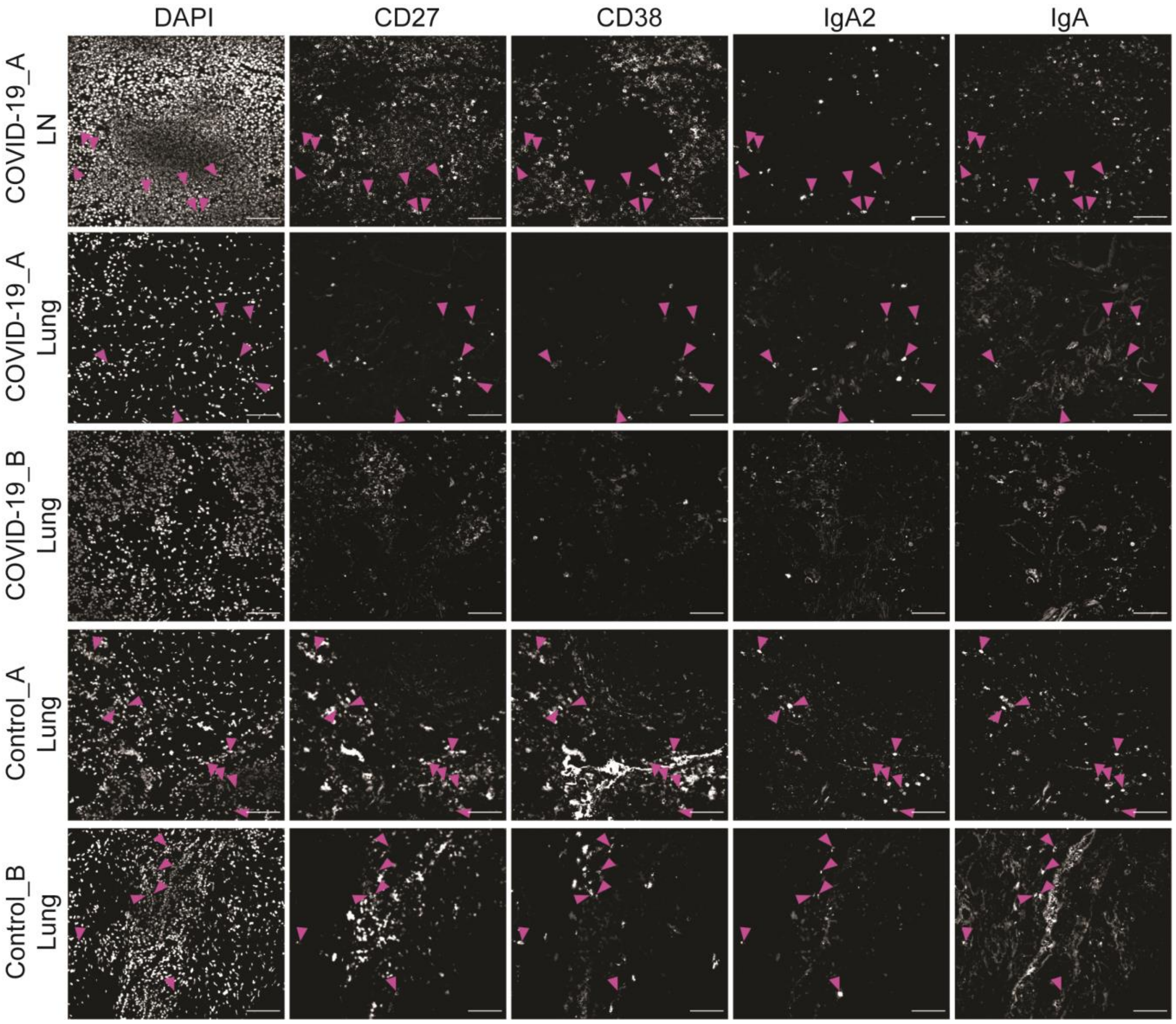

Supplementary Figure 6. IgA-expressing plasmablasts and/or plasma cells in the lungs of deceased COVID-19 patients and COVID-19-negative controls. 5-marker MELC panel acquired in SARS-CoV2-positive lymph node and lung (COVID-19_A without and COVID-19_B with sepsis) and control lungs (SARS-CoV2 negative, bacterial infection due to aspiration pneumonia). Each image depicts the same field of view, sequentially stained with the depicted fluorescence-labelled antibodies and the nuclear stain DAPI. Magenta arrows point to $IgA2^+IgA^+CD27^+CD38^+$ cells (containing a nucleus). Images are representative of two fields of view per patient, except for COVID-19_A with four fields of view analyzed and Control_B with one single field of view analyzed. Images contain 2048 x 2048 pixels and are generated using an inverted wide-field fluorescence microscope with a 20x objective, a lateral resolution of 325 nm and an axial resolution above 5 µm. Scale bar: 100 µm.

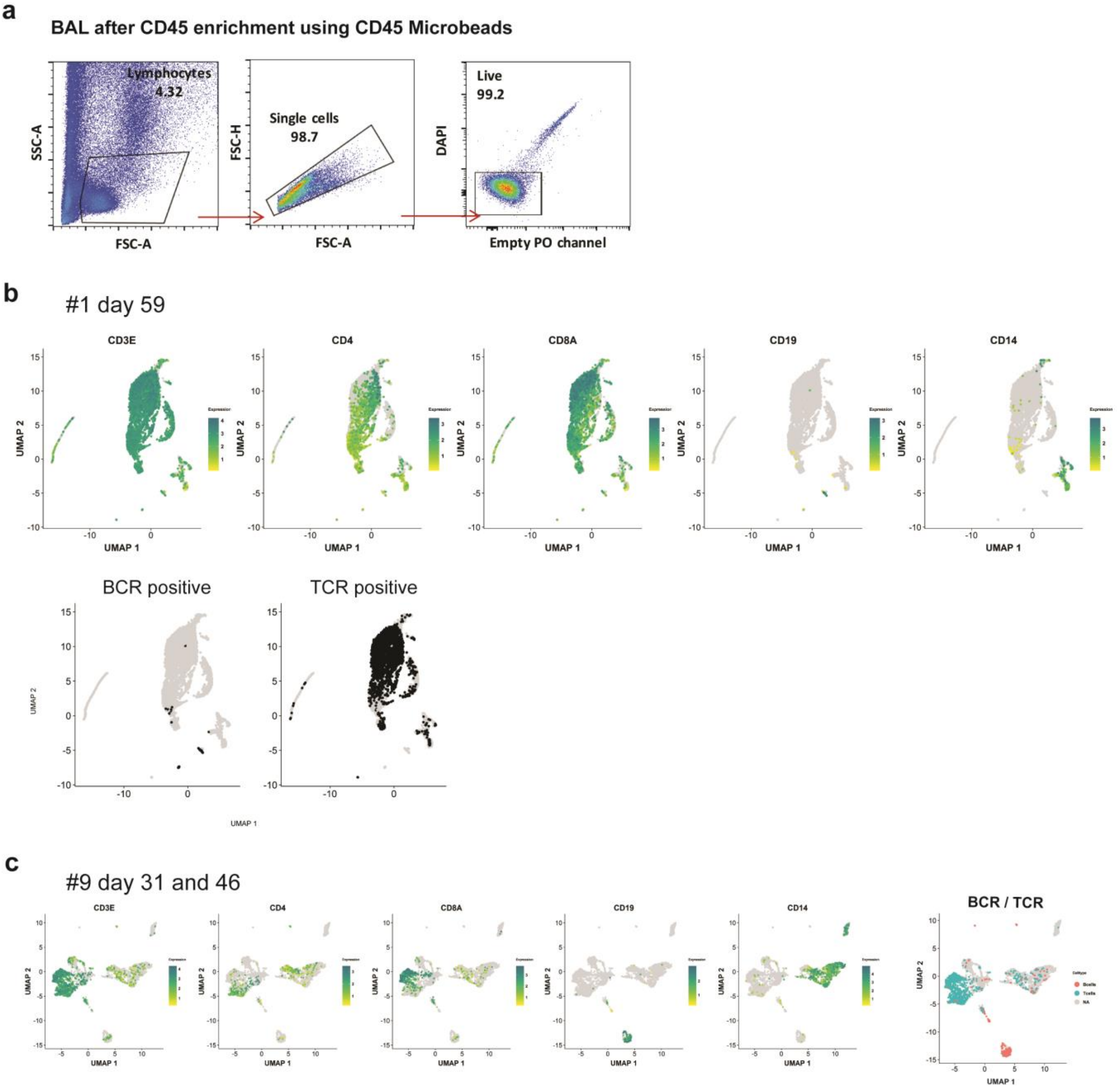

Supplementary Figure 7. Single cell transcriptomes of bronchoalveolar lavage (BAL) cells from ICU COVID-19 patients. a) Gating strategy used for FACS of live $CD45^{+}$ cells from the BAL of COVID-19 patients depicted in Figure 7. Before FACS, cells were pre-enriched by MACS using CD45 MicroBeads. **b)** UMAP representation of the expression of selected genes in the cells obtained from the BAL of patient #1 on day 59 following ICU admission (see Fig. 4d). Selected genes and BCR/TCR presence allow the identification of cell type belonging to each cluster **c)** UMAP representation of the expression of selected genes in the cells obtained from the BAL of patient #9 on days 31 and 46 following ICU admission (see Fig. 4e). Selected genes allow the identification of the cell types belonging to each cluster.

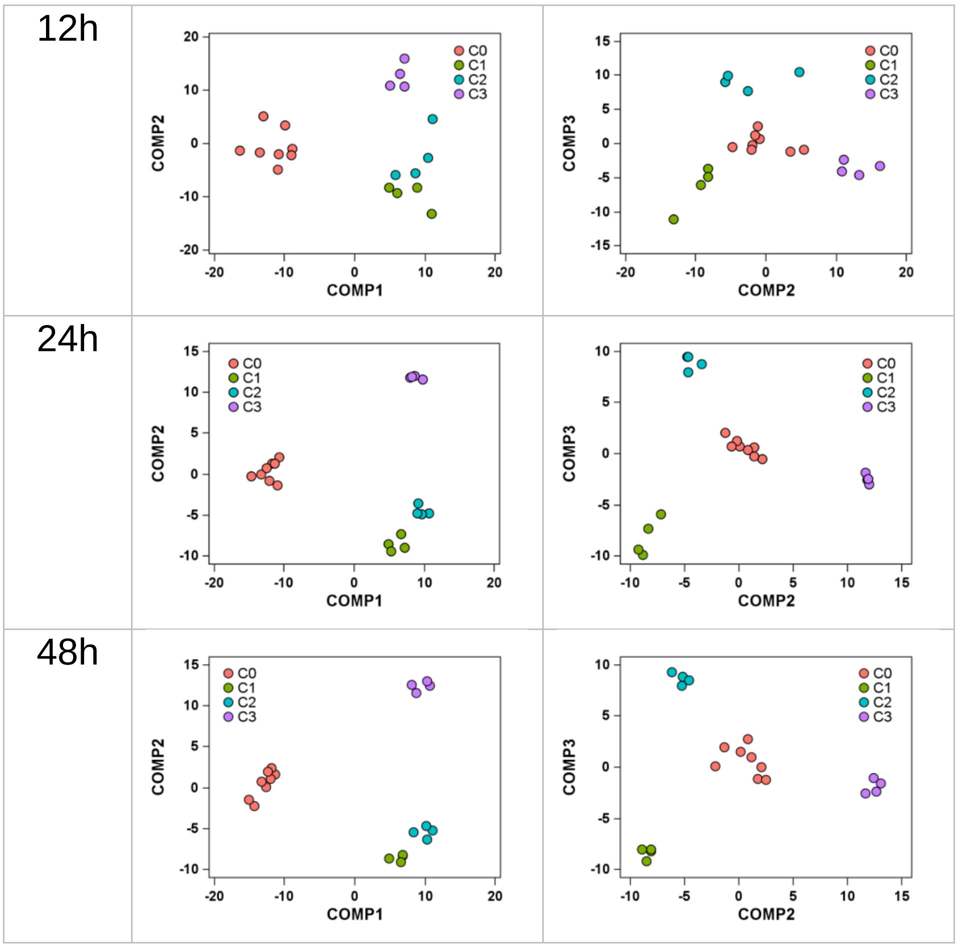

Supplementary Figure 8. Sparse Partial Least Squares Discriminant Analysis (sPLS-DA). sPLS-DA for IL2/IL21-activated B cells at day 6 (C0) and additional treatment with IL6/IL21/IFN I (C1), IL6/IL21/TGF-β (C2) and IL6/IL21/IFN I/TGF-β (C3) for 12h, 24h and 48h.

Supplementary Table 1. Demographics, disease history and serological information about S, its RBD domain and NP of SARS-CoV-2 of patients and healthy controls analyzed on Figures 1 to 3 and 7.

Patient ID	sex	age	days after ICU admission at time of analysis	days after start of symptoms at time of analysis	other viral infections	other bacterial infections	mortality	type 2 diabetes mellitus	choronic heart failure	coronary heart disease	spike protein-specific antibodies	RBD-specific antibodies	nucleoprotein-specific antibodies
HC1	m	42	N/A	N/A	n	n	n	n	n	n	n	n	n
HC2	f	55	N/A	N/A	n	n	n	n	n	n	n	n	n
HC3	m	52	N/A	N/A	n	n	n	n	n	n	n	n	n
HC4	m	72	N/A	N/A	n	n	n	n	n	n	n	N/A	N/A
1	m	76	4 / 59	not available	n	y	n	n	n	y	n / y	n / y	n / y
2	f	91	5	12	n	n	n	n	y	y	n	n	n
3	f	58	3 / 9	not available	n	n	n	y	n	n	n / y	n / y	n / y
4	f	76	9	13	n	n	n	n	n	n	y	y	y
5	m	72	9	23	n	y	n	n	n	y	y	y	y
6	m	79	12	19	n	y	y	n	n	n	y	y	y
7	m	69	13	19	n	y	n	y	n	n	y	y	y
8	f	83	3 / 18	13 / 28	n	y	n	y	n	y	n / y	n / y	n / y
9	m	76	31 / 46	36 / 51	n	y	n	n	n	n	y / y	y / y	y / y
10	f	77	5	15	n	y	y	y	n	n	n	n	n
11	f	76	7	36	n	y	n	n	n	n	y	y	y

Supplementary Table 2. Demographics, disease history and serological information about S, its RBD domain and NP of SARS-CoV-2 of patients and healthy controls analyzed on Figures 4 and 5.

Patient ID	sex	age	days after ICU admission at time of analysis	days after start of symptoms at time of analysis	other viral infections	other bacterial infections	mortality	type 2 diabetes mellitus	choronic heart failure	coronary heart disease	spike protein-specific antibodies	RBD-specific antibodies	nucleoprotein-specific antibodies
Healthy	m	40	N/A	N/A	n	n	n	n	n	n	N/A	N/A	N/A
10	f	77	13	24	n	y	y	y	n	n	y	y	y
13	m	50	29	35	n	y	n	n	n	y	y	y	y
14	m	76	32	54	n	y	n	n	y	y	y	y	y
Measles	m	38	N/A	N/A	n	n	n	n	n	n	N/A	N/A	N/A

Supplementary Table 3. Demographics and disease history of patients and controls of histologically analyzed autopsy cases (Figure 6).

Patient ID	sex	age	COVID-19 duration in days	SARS-CoV-2 qPCR ante-mortem	PMI in hours	ventilation	Comorbidities						lung tissue SARS-CoV-2 RNA*
							cardiovascular	respiratory	gastro-intestinal	CNS	urinary	others	
Covid-19_A	m	76	14	positive	30	no	arterial hypertension, ischemic cardiomyopathy, myocardial infarction (2008)	heavy smoker	–	–	–	–	6,73
Covid-19_B	f	68	34	positive	16	yes	arterial hypertension, coronary heart disease	COPD, stage 2 (Gold classification)	–	–	–	class 1 obesity, hypothyroidism	1,25
Covid-19_C	f	56	27	positive	36	yes	cardiomyopathy	-	acute liver failure	delirium	acute renal failure	class 1 obesity, type II Diabetes	2,46
Control_A	f	94	N/A	negative	60	no	coronary arteriosclerosis	bronchopneumonia due to aspiration	–	unknown neurodegenerative disease with dementia	–		N/A
Control_B	f	53	N/A	negative	48	no	–	brochopneumonia due to aspiration	–	unknown neurodegenerative disease with dementia	–	cachexia	N/A
Control_C	m	59	N/A	negative	48	no	lung emboly	bronchopneumonia due to aspiration	malnutrition	meningitis, hydrocephalus	–	–	N/A

***** in log10 copies/10,000 cells
