# Supplementary material for: SARS-CoV-2 in severe COVID-19 induces a TGF-β-dominated chronic immune response that does not target itself
Source: Nat Commun. 2021 Mar 30;12:1961. doi: 10.1038/s41467-021-22210-3 (PMC8010106; doi:10.1038/s41467-021-22210-3)
Supplement: Supplementary file 3 — Reporting Summary [file 41467_2021_22210_MOESM3_ESM.pdf]

nature research

Corresponding author(s): Mir-Farzin Mashreghi

Last updated by author(s): Feb 18, 2021

Reporting Summary

Nature Research wishes to improve the reproducibility of the work that we publish. This form provides structure for consistency and transparency in reporting. For further information on Nature Research policies, see our Editorial Policies and the Editorial Policy Checklist.

Statistics

For all statistical analyses, confirm that the following items are present in the figure legend, table legend, main text, or Methods section.

n/a	Confirmed	
☐	☒	The exact sample size (*n*) for each experimental group/condition, given as a discrete number and unit of measurement
☐	☒	A statement on whether measurements were taken from distinct samples or whether the same sample was measured repeatedly
☐	☒	The statistical test(s) used AND whether they are one- or two-sided<br>*Only common tests should be described solely by name; describe more complex techniques in the Methods section.*
☒	☐	A description of all covariates tested
☐	☒	A description of any assumptions or corrections, such as tests of normality and adjustment for multiple comparisons
☐	☒	A full description of the statistical parameters including central tendency (e.g. means) or other basic estimates (e.g. regression coefficient) AND variation (e.g. standard deviation) or associated estimates of uncertainty (e.g. confidence intervals)
☒	☐	For null hypothesis testing, the test statistic (e.g. *F*, *t*, *r*) with confidence intervals, effect sizes, degrees of freedom and *P* value noted<br>*Give P values as exact values whenever suitable.*
☒	☐	For Bayesian analysis, information on the choice of priors and Markov chain Monte Carlo settings
☒	☐	For hierarchical and complex designs, identification of the appropriate level for tests and full reporting of outcomes
☐	☒	Estimates of effect sizes (e.g. Cohen's *d*, Pearson's *r*), indicating how they were calculated

Our web collection on statistics for biologists contains articles on many of the points above.

Software and code

Policy information about availability of computer code

Data collection	Cell sorting was performed using MA900 Multi-Application Cell Sorter software. Flow cytometry data was acquired using the software BD FACSDiva (version 8.0.1, BD Biosciences)
Data analysis	Flow cytometry data was analysed using FlowJo (version 10.6.1, Tree Star). Statistical analysis was performed using GraphPad - Prism (version 5.0 and 9.0).<br>Raw sequence reads were processed using cellranger (version 3.1.0). Statistics and data analysis was performed in R (version 3.6.1) using the Seurat package (version 3.1.1). Refdata-cellranger-hg19-1.2.0 and refdata-cellranger-vdj-GRCh38-alts-ensembl-2.0.0 were used as reference.<br>Image pre-processing was done using ImageJ (version 1.47t).

For manuscripts utilizing custom algorithms or software that are central to the research but not yet described in published literature, software must be made available to editors and reviewers. We strongly encourage code deposition in a community repository (e.g. GitHub). See the Nature Research guidelines for submitting code & software for further information.

Data

Policy information about availability of data

All manuscripts must include a data availability statement. This statement should provide the following information, where applicable:

- Accession codes, unique identifiers, or web links for publicly available datasets
- A list of figures that have associated raw data
- A description of any restrictions on data availability

Data used for the study is available in Gene Expression Omnibus (GEO) under the accession code GSE158038.

Field-specific reporting

Please select the one below that is the best fit for your research. If you are not sure, read the appropriate sections before making your selection.

- [x] Life sciences
- [ ] Behavioural & social sciences
- [ ] Ecological, evolutionary & environmental sciences

For a reference copy of the document with all sections, see nature.com/documents/nr-reporting-summary-flat.pdf

Life sciences study design

All studies must disclose on these points even when the disclosure is negative.

Sample size	Sample size was determined by the availability of patient material at the time of study.
Data exclusions	No data was excluded.
Replication	Each sample was analyzed independently. We report consistent results from the analysis of different patients at each analyzed time point after ICU admission. The same is true for the analyzed controls.
Randomization	Not applicable as each patient/healthy control sample was analyzed independently upon collection/reception.
Blinding	Blinding was not possible due to the nature of the samples analyzed. Patient samples and healthy controls were analyzed independently, as patients were recruited in the intensive care unit and healthy controls not in a health care context.

Reporting for specific materials, systems and methods

We require information from authors about some types of materials, experimental systems and methods used in many studies. Here, indicate whether each material, system or method listed is relevant to your study. If you are not sure if a list item applies to your research, read the appropriate section before selecting a response.

Materials & experimental systems

n/a	Involved in the study	
[ ]	[x]	Antibodies
[ ]	[x]	Eukaryotic cell lines
[x]	[ ]	Palaeontology and archaeology
[x]	[ ]	Animals and other organisms
[ ]	[x]	Human research participants
[x]	[ ]	Clinical data
[x]	[ ]	Dual use research of concern

Methods

n/a	Involved in the study	
[x]	[ ]	ChIP-seq
[ ]	[x]	Flow cytometry
[x]	[ ]	MRI-based neuroimaging

Antibodies

Antibodies used

Anti-human antibodies
Flow Cytometry: CD27 (MT271, PE, Miltenyi Biotec, Cat No 130-097-926), CD3 (BW264/56, VioBlue, Miltenyi Biotec, Cat No 130-113-133 and OKT3, BV785, BioLegend, Cat No 317330), CD14 (TÜK4, VioBlue, Miltenyi Biotec, Cat No 130-113-152 and TM1, Pacific Orange, DRFZ in-house conjugation), CD16 (REA423, VioBlue, Miltenyi Biotec, Cat No 130-113-958), CD38 (HIT2, APC, BioLegend, Cat No 303510), CD19 (LT19, Miltenyi Biotec, Cat No 130-113-728, 1:400), CD38 (IB6, PE-Vio770, Miltenyi Biotec, Cat No 130-113-990, 1:200), CD4 (SK3, PE-Cy5.5, BioLegend, Cat No 35-0047-42), CD19 (H1B19, V500, BD Biosciences, Cat No 561121), CD137 (4B4-1, PE, Miltenyi Biotec, Cat No 130-093-476), CD154 (5C8, biotin, Miltenyi Biotec, Cat No 5190204135 and 24-31, BV421, BioLegend Cat No 310824), CD45RA (HI100, BV605, BioLegend Cat No 304133), CCR7 (G043H7, A488, BioLegend Cat No 353206), CD69 (FN50, PE-CF594, BD Biosciences, Cat No 5049599), HLA-DR (L243, APC-Cy7, Biolegend, Cat No 307617), IFNγ (4S.B3, PE-Cy7, BioLegend Cat No 502528), IL-2 (MQ1-17H12, APC-Cy7, BioLegend Cat No 500342), TNFα (Mab11, APC, BD Biosciences Cat No 554514), IgA (polyclonal, FITC, Southern Biotech, Cat No 2052-02), IgG Fc (polyclonal, Alexa647, Southern Biotech, Cat No 2014-31), IgM Fc (CH2, PE, Invitrogen, Cat No MA1-10381), IgA2 (A9604D2, Alexa647, Southern Biotech, Cat No 9140-31) and IgA1(B3506B4, Alexa488, Southern Biotech, Cat No 9130-30).
Cite-Seq: All BioLegend. CD154 (24-31, Cat No 310849), CD127 (A019D5, Cat No 351356), CD45RA (HI100, Cat No 310951), CD45RO (UCHL1, Cat No 304163), CD69 (FN50, Cat No 304259), CD39 (A1, Cat No 328237), HLA-DR (L243, Cat No 307663), CD279 (EH12.2H7, Cat No 329963), CD57 (QA17A04, Cat No 393321), CD27 (MIH26, Cat No 305651) and CD95 (DX2, Cat No 302853).
ELISA: All antibodies are alkaline phosphatase (AP)-conjugated. IgA1 (B3506B4, Southern Biotech, Cat No 9130-04), IgA2 (A9604D2, Southern Biotech, Cat No 9140-04), IgG (polyclonal, MP biomedicals, Cat No 59289) , IgM (polyclonal, Sigma-Aldrich, Cat No A3437-.25ML)
MELC: All antibodies are PE-conjugated. IgA2 (REA995, Miltenyi, Cat. No.130-117-763), CD27 (REA499, Miltenyi, Cat. No. 130-114-166); CD38 (IB6, Miltenyi, Cat. No. 130-113-427) and IgA (Clone IS11-8E10, Miltenyi, Cat. No. 130-114-002).

Non anti-human antibody

	recombinant anti-SARS-CoV-2 Spike Glycoprotein S1 antibody (CR3022, Abcam, Cat No ab273073)
Validation	All purchased antibodies were validated by their manufacturers and further in-house testing.<br>- Miltenyi Biotec, https://www.miltenyibiotec.com/DE-en/lp/antibody-validation-improved-reproducibility.html<br>In the past years, there has been a growing concern in the scientific community regarding reproducibility of experiments. This has been reflected in multiple publications and articles. In this context, antibodies are considered as one of the key sources of inconsistency in results. As a manufacturer of antibodies, we take this topic very seriously and see our responsibility in serving the scientific community with validated and consistent tools. Three pillars of antibody validation: 1. Antibody reproducibility and consistency (Pure antibody products & Lot-to-lo consistent performance); 2. Antibody specificity (Epitope competition assay, Knockout validation via targeted genome editing & RNAi knockdown); 3. Antibody sensitivity (Functional testing of every product prior to release, Performance comparison & Compatibility with fixation).<br>- BioLegend, https://www.biolegend.com/en-us/reproducibility<br>The reproducibility of published research has emerged as an urgent topic in today's scientific community. From funding agencies to researchers to manufacturers and publishers, it is critical for all of these groups to align themselves to ensure that research is done with rigor and is reproducible. How has BioLegend stepped up to meet these needs? In addition to sponsoring and collaborating with the Global Biological Standards Institute (GBSI) on setting antibody standards, we undertake extensive measures to ensure quality products that meet reproducibility requirements today and into the future.<br>BioLegend spends a considerable amount of effort in creating new antibodies for research. The majority of these new antibody products are monoclonal antibodies (mAb) produced from hybridomas. Clones of these hybridomas are carefully selected based on a number of criteria including robust growth and efficient production of a single clone of antibody that is specific to the intended target. The best clones move on to applications testing. All newly developed clones at BioLegend undergo validation testing for multiple applications. This serves as a cross-check for specificity and provides clarity for research uses. Maintaining lot-to-lot consistency is vital for reproducibility. It simply is not enough for antibody manufacturers to validate antibodies just once. Pass/fail specification requirements are essential for quality control testing of every lot of product. BioLegend maintains records for all lot-specific testing<br>- BD Biosciences, https://www.biocompare.com/Antibody-Manufacturing/355107-Antibody-Manufacturing-Perspectives-BD-Bioscience/<br>We conduct quality control (QC) testing in primary model systems to ensure biological accuracy in an ISO 9001 certified facility. BD carefully selects and characterizes antibody content in product development and tests in relevant primary model systems to ensure biological accuracy. BD conducts rigorous QC testing of each antibody lot tested side-by-side with a previously produced lot as reference. Our product development process includes testing on a combination of primary cells, cell lines and/or transfectant cell models with relevant controls using multiple immunoassays to ensure biological accuracy. We also perform multiplexing with additional antibodies to interrogate antibody staining in multiple cell populations. BD believes antibody validation is critical to ensure accurate scientific results. Both the consumer and the reagent provider share the responsibility for reproducible science.<br>- Abcam, https://www.abcam.com/primary-antibodies/how-we-validate-our-antibodies<br>To achieve accurate and precise results, you need antibodies that consistently bind specifically and selectively to the intended target. Antibody validation must be application-specific to be effective. ELISA: We validate the performance of each antibody pair in plasma, serum, or tissue lysates, using spike-recovery experiments to validate antibody selectivity. This method involves 'spiking' purified recombinant target proteins into the biological matrix, which should be recovered and detected within -/+ 20% variation (80–120%) of the kit's expected protein standard signal in the provided diluent. The recovery observed for the spike should be almost identical in both the biological matrix and the standard diluent for a sample matrix to be considered valid for our ELISA kits.Linearity studies of sample dilutions are also carried out using sandwich ELISA to ensure that our antibody pairs recognize not only purified recombinant protein, but also native target protein. To further verify antibody specificity for use in ELISA, we test to what degree the antibodies bind to related proteins or family members. This allows us to gauge any cross-reactivity and interference. Acceptable results for us will show less than 5% cross-reactivity.<br>- Invitrogen, https://www.thermofisher.com/de/de/home/life-science/antibodies/invitrogen-antibody-validation.html<br>Antibodies are some of the most critical research reagents used in the lab. Poor specificity or application performance can significantly frustrate the ability to obtain good results, which can cause critical delays. Underperforming antibodies result in a lack of reproducibility, wasting time and money. In other words, researchers need antibodies that bind to the right target and work in their applications every time. To help ensure superior antibody results, we've expanded our specificity testing methodology using a 2-part approach for advanced verification. Part 1—Target specificity verification. This helps ensure the antibody will bind to the correct target. Our antibodies are being tested using at least 1 of the following methods to ensure proper functionality in researcher's experiments. Part 2—Functional application validation. These tests help ensure the antibody works in a particular application(s) of interest.

Eukaryotic cell lines

Policy information about cell lines

Cell line source(s)	HEK293T cells from ATCC (293T (ATCC® CRL-3216™)), Vero E6 (ATCC® CRL-1586™)
Authentication	From vendor. ATCC authenticates cell lines routinely by STR profiling, cellular morphology, karyotyping and cytochrome C oxidase I assay.
Mycoplasma contamination	Cells were not tested for mycoplasma contamination during the time of the study.
Commonly misidentified lines<br>(See ICLAC register)	No commonly misidentified lines were used in this study.

Human research participants

Policy information about studies involving human research participants

Population characteristics	Healthy control 1, male, age 42<br>Healthy control 2, female, age 55<br>Healthy control 3, male age 52<br>Healthy control 4, male, age 72<br>Healthy control 5, male, age 40 (T cell scRNA-Seq)<br>Healthy control 6, male, age 38 (Tcell scRNA-Seq, measels)<br>Patient 1, male, age 76, analyzed at day 4 and 59 after ICU admission, bacterial infection, coronary heart disease<br>Patient 2, female, age 91, analyzed at day 5 after ICU admission, chronic heart failure, coronary heart disease<br>Patient 3, female, age 58, analyzed at day 3 and 9 after ICU admission, type 2 diabetes mellitus<br>Patient 4, female, age 76, analyzed at day 9 after ICU admission<br>Patient 5, male, age 72, analyzed at day 9 after ICU admission, bacterial infection, coronary heart disease<br>Patient 6, male, age 79, analyzed at day 12 after ICU admission, bacterial infection, deceased<br>Patient 7, male, age 69, analyzed at day 13 after ICU admission, bacterial infection, type 2 diabetes mellitus<br>Patient 8, female, age 83, analyzed at day 3 and 18 after ICU admission, bacterial infection, type 2 diabetes mellitus, coronary heart disease<br>Patient 9, male, age 76, analyzed at day 31 and 46 after ICU admission, bacterial infection<br>Patient 10, female, age 77, analyzed at day 5 and 13 after ICU admission, bacterial infection, type 2 diabetes mellitus, deceased<br>Patient 13, male, age 50, analyzed at day 28 after ICU admission, bacterial infection, coronary heart disease<br>Patient 14, male, age 76, analyzed at day 32 after ICU admission, bacterial infection, chronic heart failure, coronary heart disease<br><br>Autopsies<br>COVID-19_A, male, age 76, 14 days of COVID-19 duration, PMI 30 hours, cardiovascular disease<br>COVID-19_B, female, age 68, 34 days of COVID-19 duration, PMI 16 hours, cardiovascular and respiratory disease, Class 1 obesity and hypothyroidism<br>COVID-19_C, female, age 56, 27 days of COVID-19 duration,PMI 36 hours, cardiomyopathy, acute liver failure, delirium, acute reanl failure, class 1 obesity<br>Control_A, female, age 94, PMI 60 hours, bronchopneumonia due to aspiration, cardiovascular disease, unknown neurodegenerative disease with dementia<br>Control_B, female, age 53, PMI 60 hours, bronchopneumonia due to aspiration, unknown neurodegenerative disease with dementia, cachexia<br>Control_C, male, age 59, PMI 48 hours, lung emboly, bronchopneumonia due to aspiration, malnurition, meningitis, hydrocephalus
Recruitment	All patients were recruited from the Intensive Care Unit of the Charité Campus Benjamin Franklin. Their inclusion in the study was based on documented disease history and SARS-CoV-2 infection that was verified by the levels of antigen-reactive antibody IgG titers. Healthy controls were recruited at the DRFZ Berlin according to their serology (negative for SARS-CoV-2-binding antibodies) and age. Autopsies were performed at the Department of Pathology and Neuropathology, Charité - Universitätsmedizin Berlin.
Ethics oversight	Ethics Committee of the Charité (EA 1/144/13 with EA 1/075/19 and EA 2/066/20) in compliance with the Declaration of Helsinki.

Note that full information on the approval of the study protocol must also be provided in the manuscript.

Flow Cytometry

Plots

Confirm that:

- [x] The axis labels state the marker and fluorochrome used (e.g. CD4-FITC).
- [x] The axis scales are clearly visible. Include numbers along axes only for bottom left plot of group (a 'group' is an analysis of identical markers).
- [x] All plots are contour plots with outliers or pseudocolor plots.
- [x] A numerical value for number of cells or percentage (with statistics) is provided.

Methodology

Sample preparation	For preparation of B cells, B cells were enriched from peripheral blood using StraightFrom® Whole Blood CD19 MicroBeads (Miltenyi Biotec) according to manufacturer's instructions. Enriched cells were incubated with Fc Blocking Reagent (Milteniy Biotec) following manufacturer's instructions and subsequently stained with anti-human antibodies for FACS. Alternatively, B cells were sorted from frozen PBMCs. For preparation of T cells, isolation of SARS-CoV-reactive effector/memory and regulatory CD4+ T lymphocytes was performed as described (Okhrimenko, A, 2014, PNAS). PBMCs were stimulated for 6 h

with anti-CD28 (1µg/mL) and the mixed spike glycoprotein (S), the membrane glycoprotein (M), and the nucleocapsid phosphoprotein (N) of the SARS-CoV-2, each 1 µg/peptide/mL, in the presence of 1 µg/mL anti-CD40. Stimulated cells were enrichment by MACS for CD137+ and CD154+ cells in two consecutive MS columns (Miltenyi Biotec) and the enriched cells incubated with anti-human antibodies for FACS or flow cytometry analysis. For preparation of BAL cells, bronchoalveolar lavage was passed via 70 µm cell strainer and lymphoid cells were isolated using anti-human CD45 microbeads (Miltenyi Biotec) followed by FACS of live cells.

Instrument	MA900 Multi-Application Cell Sorter (Sony Biotechnology) and LSRFortessa flow cytometer (BD Biosciences)
Software	Sony MA900 software (Sony Biotechnology), FACSDiva (version 8.0.1 BD Biosciences) and FlowJo (version 10.6.1, Tree Star)
Cell population abundance	Cell population abundance was highly variable among patients and healthy controls. Sorted population purity was analyzed during post-sorting cell counting using a MACSQuant flow cytometer (Miltenyi Biotec).
Gating strategy	B cell sorting: SSC-A x FSC-A, FSC-A x FSC-H, Live/CD3-/CD14-/CD16-, CD38high x CD27high.<br>T cell sorting: SSC-A x FSC-A, FSC-H x FSC-W, Live/CD19-/CD14- x CD3+, CD4+, among CD137 x CD154 CD137+ or CD154+<br>T cell analysis: Time x SSC-A, SSC-A x FSC-A, FSC-H x FSC-W, Live/CD19-/CD14- x CD3+, CD4+, among CD137 x CD154 CD137+ or CD154+. Among CD154+, IFN-g+ or IL-2+ or TNF+.<br>BAL cell sorting: SSC-A x FSC-A, FSC-A x FSC-H, Live

☒ Tick this box to confirm that a figure exemplifying the gating strategy is provided in the Supplementary Information.
