# Supplementary material for: SARS-CoV-2 in severe COVID-19 induces a TGF-β-dominated chronic immune response that does not target itself
Source: Nat Commun. 2021 Mar 30;12:1961. doi: 10.1038/s41467-021-22210-3 (PMC8010106; doi:10.1038/s41467-021-22210-3)
Supplement: Supplementary file 4 — Description of Additional Supplementary Files [file 41467_2021_22210_MOESM4_ESM.pdf]

Description of Additional Supplementary Files

File Name: **Supplementary Data 1**

Description: **Example trees from expanded BCR clonal families.** To evaluate B cell receptor (BCR) clonality and hypermutation in severe COVID-19 patients, single cell BCR repertoires from sorted CD38^{high} CD27^{high} B cells from severe COVID-19 patients and healthy controls were sequenced and analyzed. Some example trees from expanded BCR clonal families are shown for each in-depth-analyzed patient/healthy control (one tree per sheet in the file). For hypermutation analysis, clonal families were defined by the same VJ-gene usage, gapped germline FR1-FR3 sequence and the nucleotide CDR3 sequence length of the heavy and light chain. Hypermutation trees were computed using GLaMST with concatenated FR1-FR3 sequences of the heavy and light chain and the germline sequence as root input. Used abbreviations: Cf, Clonal family; Pat, Patient; HC, Healthy Control.
